# Japanese Patients With Obesity-Related Bronchial Asthma May Benefit From Laparoscopic Sleeve Gastrectomy

**DOI:** 10.7759/cureus.93265

**Published:** 2025-09-26

**Authors:** Mitsuhiro Sumitani, Seiichi Kitahama, Akiko Oka, Yasuyuki Iwahashi, Shinsuke Nakajima

**Affiliations:** 1 Department of Respiratory and Sleep Medicine, Center for Obesity, Diabetes and Endocrinology, Chibune General Hospital, Osaka, JPN; 2 Department of Surgery, Kyoto University Hospital, Kyoto, JPN; 3 Department of Metabolic and Bariatric Surgery, Center for Obesity, Diabetes and Endocrinology, Chibune General Hospital, Osaka, JPN; 4 Department of Diabetes, Metabolism and Endocrinology, Center for Obesity, Diabetes and Endocrinology, Chibune General Hospital, Osaka, JPN

**Keywords:** bariatric surgery, bronchial asthma, insulin resistance, laparoscopic sleeve gastrectomy, obesity, obesity-related bronchial asthma

## Abstract

Background: Weight loss can effectively improve asthma control for some patients with obesity-related bronchial asthma, but it is difficult in certain patients. Bariatric surgery may enhance asthma control in such patients.

Objectives: We evaluate the effects of laparoscopic sleeve gastrectomy (LSG), as an example of bariatric surgery, on asthma control in patients with obesity-related bronchial asthma.

Methods: We analyzed 521 of our patients who underwent bariatric surgery between June 2016 and February 2024. They each had a self-reported history of bronchial asthma and were receiving treatment for it at the time of surgery. We assessed Asthma Control Test (ACT) scores, short-acting beta agonist (SABA) use based on the ACT, and respiratory function tests before and after surgery. Statistical analysis was performed using Wilcoxon's signed rank test, with significance defined as P < 0.05.

Results: Fifteen patients were included (86.7% female, median age: 46 years, median body mass index (BMI): 41.0 kg/m², median ACT score: 22). Fourteen of them underwent only LSG; the other also had duodenal bypass. The median percentage total weight loss at six months post surgery was 21.8%. ACT scores improved from 20.8 ± 3.5 to 24.4 ± 1.2 points (P = 0.0039), forced expiratory volume in 1 second (FEV1) increased from 2.45 ± 0.47 L to 2.74 ± 0.51 L (P = 0.0001), and SABA use decreased by 86.7%, indicating that the patients had better bronchial asthma control than before surgery.

Conclusion: LSG appears to improve asthma control in certain patients with obesity-related bronchial asthma.

## Introduction

Obesity is recognized as one of the phenotypes of difficult-to-treat bronchial asthma and has been categorized based on its association with body mass index (BMI) [1]. The impact of obesity on asthma is multifactorial, involving mechanical restriction, altered airway inflammation, and metabolic dysfunction [2,3].

Meanwhile, we suggest that there may be a difference in the impact of obesity on asthma between Japanese and Western populations. In Japanese individuals, asthma exacerbation reportedly becomes more frequent at a BMI ≥25 kg/m², rather than the larger Western obesity threshold of ≥30 kg/m² [4,5], and a higher asthma prevalence has also been reported in this population [6]. This lower threshold reflects ethnic differences in body composition, fat distribution, and susceptibility to metabolic dysfunction [7,8].

Previous studies have demonstrated improvement in asthma control with ≥10% weight loss [9]. However, weight loss interventions are often very difficult for patients with severe obesity who are unable to exercise. As for bronchial asthma associated with severe obesity, improvement of bronchial asthma symptoms can be achieved by weight loss intervention through laparoscopic gastric banding surgery [10]. Bariatric surgery, including laparoscopic sleeve gastrectomy (LSG), may represent a feasible intervention. Nevertheless, data on Japanese patients are extremely limited. Therefore, this study aimed to evaluate the effects of LSG on asthma control among Japanese patients with obesity-related asthma.

This article was previously presented as a meeting abstract at The 26^th^ World Congress of the International Federation for the Surgery of Obesity and Metabolic Disorders (IFSO) on September 4^th^, 2024.

## Materials and methods

Study design and methods

This was a single-center retrospective cohort study conducted at Chibune General Hospital, Osaka, Japan. We analyzed patients who underwent bariatric surgery for severe obesity between June 2016 and February 2024 at our hospital. Among the patients considered, those who had reported a history of asthma in the preoperative questionnaire were screened for eligibility. Patients were selected based on the following criteria: age ≥18 years, ongoing bronchial asthma treatment at the time of surgery, and undergoing bariatric surgery for the first time. Exclusion criteria included pacemaker use, smokers with a Brinkmann index of ≥600, severe mental illness, and secondary obesity.

All procedures adhered to the Declaration of Helsinki, with approval of Chibune Hospital's Institutional Review Board (IRB) (approval number: A202403), and patients were informed about the study via the hospital website, with consent obtained on an opt-out basis.

Clinical data, definitions, and outcomes

Data collection included demographics, Asthma Control Test (ACT) scores, pulmonary function tests (percentage forced vital capacity (%FVC), forced expiratory volume in 1 second (FEV1), and expiratory reserve volume (ERV)), and short-acting beta-agonists (SABA) use. The definition of asthma was based on the Global Initiative for Asthma (GINA) guidelines [11]. The ACT assessment and pulmonary function tests were conducted during outpatient follow-up visits post surgery, with the ACT assessment performed six months postoperatively.

Timing of postoperative assessments

Both ACT and pulmonary function tests were planned six months after surgery. In this cohort, the observed timing was six months (6.0-6.0) for ACT (n=10) and six months (6.0-8.0) for pulmonary function tests (n=15); when ACT was not obtained at the planned visit, we used the nearest routine postoperative visit for pulmonary function tests. Prior to pulmonary function testing, all patients received long-acting beta-agonists, and the pulmonary function values measured under SABA-free conditions were used for preoperative evaluations. Postoperative pulmonary function values were also obtained under SABA-free conditions. Additionally, fractional exhaled nitric oxide (FeNO) testing was not performed in any of the patients preoperatively. Permission to use the ACT was obtained from GlaxoSmithKline (London, United Kingdom).

Eligibility criteria

The primary endpoint was preoperative and postoperative change in the ACT score, with an improvement of ≥3 points, which was recognized as the minimally important difference (MID) for ACT, indicating an improvement in bronchial asthma [12]. However, cases with a preoperative ACT score of ≥23 and that could not achieve a MID of ≥3 were analyzed separately. Secondary endpoints included preoperative to postoperative changes in respiratory function (%FVC, FEV1, and ERV) and the number of times of rescue SABA use evaluated based on ACT score data. The percentage of excess weight loss (%EWL) was calculated as follows:

\[ \% \text{Excess Weight Loss (EWL)} = \left( \frac{\text{Weight Loss}}{\text{Actual Body Weight} - \text{Ideal Body Weight}} \right) \times 100 \]

Statistical analysis

For continuous variables, data are presented as medians with interquartile ranges (IQRs). Because this study comprises paired pre-post measurements from the same individuals and the sample size is small, we prespecified non-parametric within-subject methods and did not use paired t-tests. Paired pre- and postoperative values were compared using the Wilcoxon signed-rank test. Zero differences were excluded from the test statistic (Wilcoxon method; not the Pratt variant). Two-sided p-values were calculated using the large-sample normal approximation with continuity correction; ties in absolute differences were assigned average ranks. Continuous variables are presented as median (IQR), and categorical variables as n (%). Analyses were performed in JMP version 9 (SAS Institute, Cary, NC, USA). The study followed Strengthening the Reporting of OBservational Studies in Epidemiology (STROBE) guidelines, and a flow diagram of patient selection is provided (Appendix A, B).

## Results

Patient characteristics and treatment profiles of asthmatic patients undergoing LSG

Of the 521 patients who underwent initial bariatric surgery for severe obesity, 103 patients reported a history of bronchial asthma in the preoperative questionnaire, and 15 patients were being treated for bronchial asthma at the time of surgery. Patient backgrounds are shown in Table 1. Among these patients, 13 (86.7%) were women, and approximately half had a BMI > 40 kg/m². According to the GINA 2024 treatment steps, 13 patients were categorized as Step 3 and two as Step 4. Fourteen underwent LSG, and only one patient had both LSG and duodenojejunal bypass. At six months postoperatively, a time point commonly used to evaluate the effectiveness of bariatric surgery, 11 patients (73.3%) achieved a %EWL greater than 50% , which is a better predictor of clinically significant change [13].

**Table 1:** Characteristics of the 15 patients who were still on asthma therapy at the time of surgery ACT: asthma control test; AHI: apnea hypopnea index; ATH: antihistamine; BMI: body mass index; CPAP: continuous positive airway pressure; DM: diabetes mellitus; ERV: expiratory reserve volume; EWL: excess weight loss; FEV1: forced expiratory volume in 1 second; FVC: forced vital capacity; GINA: Global Initiative for Asthma; HOMA-IR: homeostasis model assessment-insulin resistance; HL: hyperlipidemia; HTN: hypertension; ICS: inhaled corticosteroids; IQR: interquartile range; LABA: long-acting β₂ agonist; LAMA: long-acting muscarinic antagonist; LTRA: leukotriene receptor antagonist; MEPO: mepolizumab; PSL: prednisolone; REI: respiratory event index; SABA: short-acting B2 agonist; SAS: sleep apnea syndrome; WBC: white blood cell count

Variable	Data
Age (median: IQR): years	46 (39.5-53.5)
Gender (male/female)	2/13
BMI (median: IQR): kg/m2	41 (36.5-52.1)
Visceral fat percentage (median: IQR): cm2	29.6 (26.8-34.9)
Leptin (median: IQR): ng/ml	41.5 (18.2-57.7)
Adiponectin (median: IQR): μg/ml	4.3 (2.6-5.3)
Insulin: μU/ml	14.9 (9.1-20.7)
HOMA-IR (median: IQR)	3.8 (2.4-5.4)
HbA1c (median: IQR): %	6 (5.8-6.8)
ABCD score: points	5 (3.5-6.5)
Complicated condition	DM:7, HL:8, HTN:8, SAS: 11
History of smoking	Non-smoker:10, Ex-smoker: 5
Asthma Control Test (ACT) score (median: IQR): points	22 (19.8-22.3)
Asthma intervention	ICS+LABA：10
	ICS+LABA+LTRA : 2
	ICS+LABA+LAMA+LTRA+PSL :1
	ICS+LABA+LTRA+MEPO : 1
	ICS+LABA+ATH: 1
GINA 2024 Step	STEP 3: 13
	STEP 4: 2
Type of phenotype on asthma	Type2:8, Non-Type2:5, unknown:2
IgE (median: IQR) :IU/ml	36.4 (32.6-510.5)
WBC count (median: IQR): /μl	7200 (5750-10150)
Eosinophil count (median: IQR) : /μl	151.5 (116.4-310.1)
% FVC (median: IQR) : %	104.5 (99.8-119.0)
FEV1 (median: IQR) : L	2.8 (2.38-3.1)
FEV1/FVC (median: IQR) : %	81.5 (78.6-84.6)
ERV (median: IQR): L	0.65 (0.47-0.83 )
AHI/REI (Median: IQR): /hr	35.6 (30.5-79.6) / 31.7 (11.2-43.8)
SAS Intervention	CPAP: 10, no treatment: 5
% EWL (6 months) (median: IQR):%	58.4 (45.9-81.6)
Surgical procedure (Sleeve/Sleeve bypass)	14/1

Changes in pulmonary function, biomarkers, and asthma control following LSG

**Figure 1:**
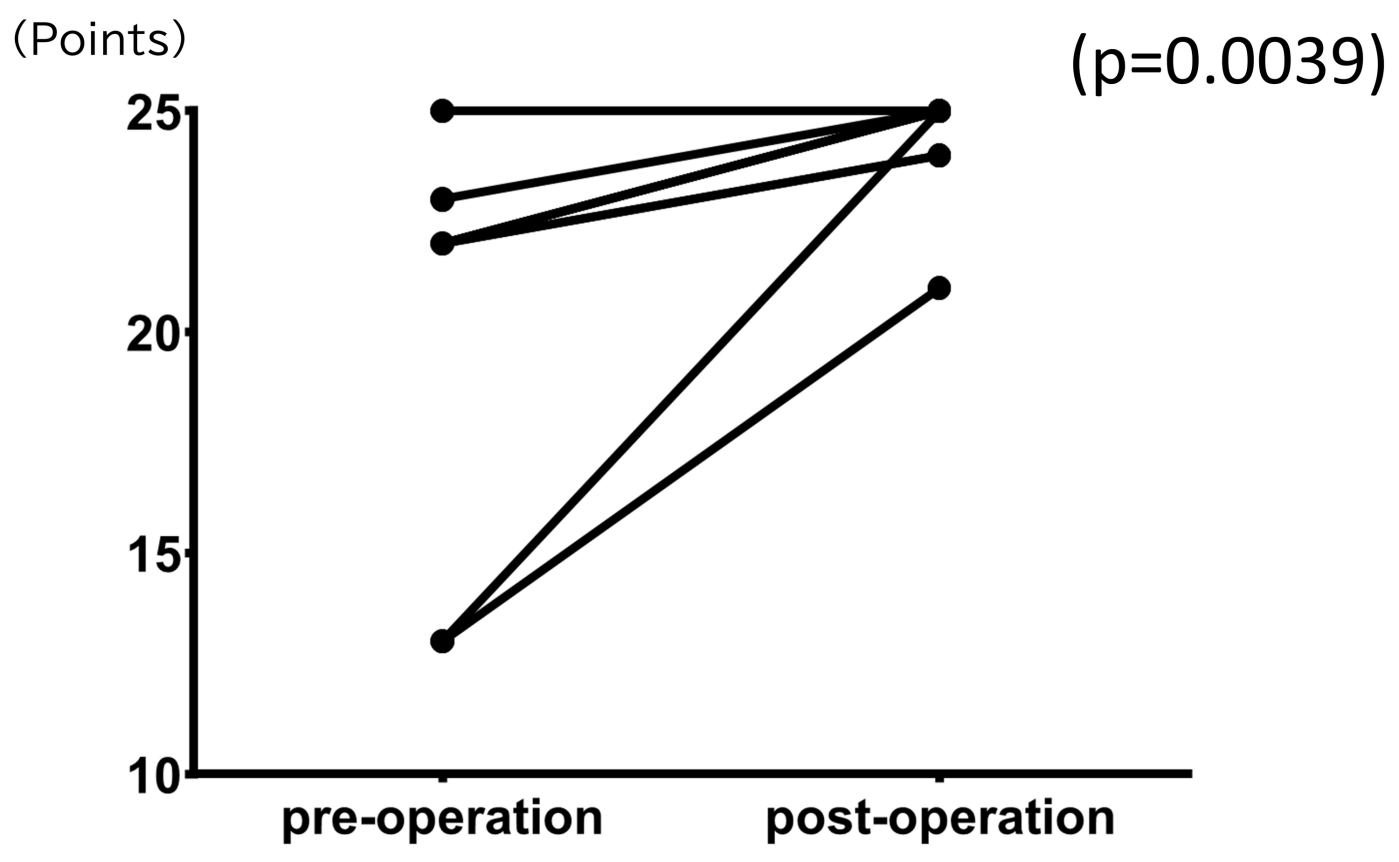
Evaluation of pre- and postoperative ACT evaluations (n=10) Following LSG, ACT increased from 22.0 (20–22.25) to 25.0 (24.0–25.0) (H-L Δ 3.0; 95% CI 2.0-3.0; p=0.0039; n=10), indicating a clinically meaningful improvement. Around 75% (6/8 patients) had an improvement of ≥3 points in ACT after surgery, except for two patients who had an ACT score of ≥23 before surgery. Wilcoxon signed-rank test; two-sided p-values (normal approximation with continuity correction); 0 differences excluded; ties averaged; n denotes the number of non-zero pairs. ACT: asthma control test

Among the 15 cases evaluated, 10 were identified in which ACT assessments were performed both before and after surgery. Following LSG, ACT increased from 22.0 (20.0-22.25) to 25.0 (24.0-25.0) (H-L Δ 3.0; 95% CI 2.0-3.0; p=0.0039; n=10), indicating a clinically meaningful improvement (Figure 1).

**Figure 2:**
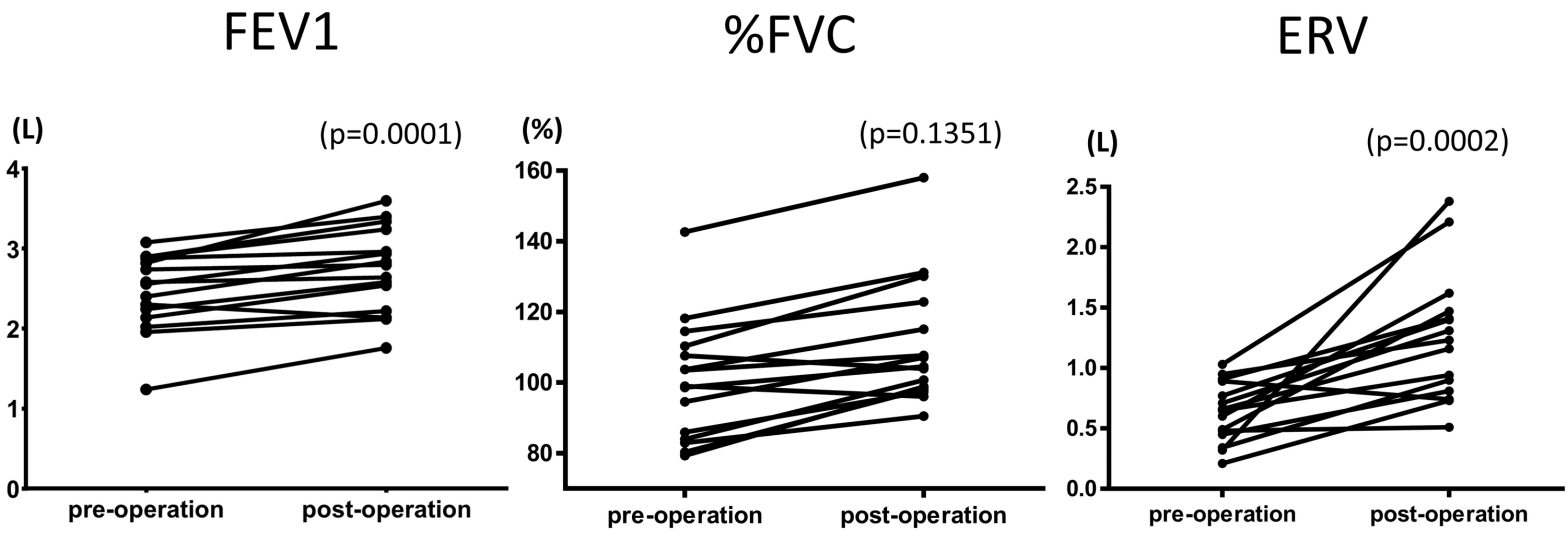
The preoperative and postoperative pulmonary function test results of 15 patients who underwent surgical intervention The parameters evaluated include FEV1 on the left, %FVC in the middle, and ERV on the right. Following LSG, FEV1 rose from 2.56 (2.14–2.88) L to 2.80 (2.22–3.24) L (H-L Δ 0.26 L; 95% CI 0.12–0.68; p=0.0001; n=15). ERV nearly doubled from 0.65 (0.45–0.89) L to 1.23 (0.81–1.47) L (H-L Δ 0.56 L; 95% CI 0.27–0.91; p=0.0002; n=15). However, %FVC did not change from 99.0 (84.0–110.3)% to 104.5 (98.2–122.8)% (H-L Δ 11.2%; 95% CI -3.0–21.6; p=0.1351; n=15). Wilcoxon signed-rank test; two-sided p-values (normal approximation with continuity correction); 0 differences excluded; ties averaged; n denotes the number of non-zero pairs. LSG: laparoscopic sleeve gastrectomy; ERV: expiratory reserve volume; %FEV1: percentage of forced expiratory volume in 1 second; FVC: forced vital capacity; PFT: pulmonary function test

In tipping-point analyses assuming no improvement (ΔACT=0) for all missing cases, the direction of effect remained unchanged, and the Hodges-Lehmann median change stayed positive. FEV1 rose from 2.56 (2.14-2.88) L to 2.80 (2.22-3.24) L (H-L Δ 0.26 L; 95% CI 0.12-0.68; p=0.0001; n=15). ERV nearly doubled from 0.65 (0.45-0.89) L to 1.23 (0.81-1.47) L (H-L Δ 0.56 L; 95% CI 0.27-0.91; p=0.0002; n=15). However, %FVC did not change from 99.0 (84.0-110.3)% to 104.5 (98.2-122.8)% (H-L Δ 11.2%; 95% CI -3.0-21.6; p=0.1351; n=15) (Figure 2).

Of the two patients with preoperative ACT scores of ≥23, one changed from 23 to 25, and the other had no change from 25. Regarding rescue SABA use, eight out of 10 patients (80％) were evaluated as not requiring SABA use at the time of postoperative evaluation (Appendices C, D). Two cases did not reach the ACT MID despite experiencing weight loss. Notably, although both patients showed postoperative increases in FEV1 and ERV, their %EWL at six months post surgery did not reach 50%, and their preoperative homeostasis model assessment-insulin resistance (HOMA-IR) values were markedly elevated (8.1 and 9.1, respectively) (Appendix E)

## Discussion

Obesity-related bronchial asthma and respiratory function assessment

This study assessed the improvement in asthma control in Japanese patients with obesity-related bronchial asthma undergoing bariatric surgery, primarily LSG, with one case involving a combination of LSG with duodenojejunal bypass. The improvements in ACT score, FEV1, and ERV suggest that LSG alleviates both mechanical and metabolic burdens contributing to asthma severity. Importantly, this is the first report focusing on Japanese patients, who are at increased risk of asthma exacerbation at lower BMI thresholds (≥25 kg/m²). This highlights the necessity of considering ethnic-specific differences in obesity-related asthma [5,8].

The ACT, a well-established symptom-based evaluation tool [14], is considered to be effective for patients with obesity-related bronchial asthma and thus served as the primary endpoint in this study. However, some patients with severe obesity may regularly experience exertional dyspnea that could be misinterpreted as asthma-related shortness of breath, potentially leading to confusion in the assessment.

Airway inflammation and insulin resistance

While lifestyle modification remains the cornerstone of obesity treatment, many patients with severe obesity cannot achieve sufficient weight loss. Our findings support LSG as a valuable therapeutic option in this population. Of note, patients with persistent insulin resistance (elevated HOMA-IR) showed limited ACT improvement despite weight loss, suggesting a potential interaction between metabolic status and asthma outcomes [15].

Weight loss and bariatric surgery as treatment for obesity-related asthma

In obesity-related bronchial asthma, weight loss has been shown to improve airway irritability, asthma control, respiratory function, and quality of life [16]. Notably, the pathophysiological relationship between obesity and asthma may differ between Western and Japanese populations. In Japanese individuals, asthma exacerbation frequency and prevalence have been reported to be higher even at BMI levels ≥25 kg/m², which is below the reported Western threshold for obesity [4,6]. This suggests that metabolic and inflammatory factors, rather than solely mechanical effects of fat accumulation, may play a more dominant role in asthma pathogenesis among not only Japanese people but also people of other ethnicities. Weight management is therefore particularly critical in this population.

Bariatric surgery for patients with obesity-related bronchial asthma is not widely reported, but all of those in the literature have noted improvements in asthma symptoms. For example, laparoscopic adjustable gastric banding has been associated with improved asthma control not only one year postoperatively but also five years postoperatively [17]. LSG, which most patients in this study underwent, is a different technique from laparoscopic adjustable gastric banding. LSG involves resection of the greater curvature of the stomach, which reduces gastric volume while also promoting hormonal changes such as increased serum glucagon-like peptide-1 levels and plasma bile acid concentrations [18]. These metabolic changes may further contribute to improved asthma control, making LSG a potentially favorable surgical option for patients with obesity-related asthma.

Interpretation

In the context of prior literature showing that ≥10% weight loss improves asthma control and lung mechanics in obesity-related asthma, our findings align with and extend those observations to a Japanese cohort. The clinically meaningful ACT gain (median H-L Δ 3.0) together with increases in FEV1 and ERV suggests that LSG may relieve obesity-related mechanical loading (e.g., diaphragmatic elevation, decreased chest wall compliance) while concurrently improving the metabolic milieu that can modulate airway inflammation. Notably, ERV nearly doubled, consistent with mitigation of obesity-related expiratory restriction and small airway closure, whereas %FVC did not materially change internal contrast that supports a mechanistic interpretation focused on air-trapping and chest wall mechanics rather than parenchymal volume change per se.

In addition, prior surgical studies (e.g., adjustable gastric banding) have reported sustained asthma control benefits; our data suggest that LSG, through both anatomical reduction and hormone/bile-acid-mediated metabolic effects, may similarly enhance asthma control in patients with severe obesity. Collectively, these results support LSG as a complementary strategy to guideline-directed pharmacotherapy in obesity-related asthma, particularly where lifestyle-only interventions have proven insufficient.

Limitation

This study has limitations. First, the sample size was small and derived from a single-center retrospective cohort, which increases the risk of selection bias and limits statistical power. Second, outcome assessments were not synchronized to a uniform postoperative time point, introducing timing heterogeneity. Third, FeNO and other type-2 inflammatory biomarkers were unavailable, precluding inflammatory endotyping and mechanistic correlation. Fourth, although within-patient pre-post analyses mitigate between-subject confounding, residual confounding remains possible (e.g., variation in inhaled corticosteroid dose, biologic use, obstructive sleep apnea (OSA)/continuous positive airway pressure (CPAP) status and adherence, and perioperative care pathways). Fifth, one case underwent LSG with duodenojejunal bypass, which may not be directly comparable to LSG alone. Finally, ACT data were complete for only a subset, and the absence of a non-surgical control group precludes causal inference.

Despite these constraints, the cohort represents real-world Japanese patients with severe obesity and active asthma treatment, a population in whom lifestyle-based weight loss is often insufficient. Given the observed improvements in ACT, FEV1, and ERV, LSG may be considered a potential therapeutic option, alongside standard pharmacologic care, for Japanese patients with obesity-related asthma who require substantial weight reduction. External validity beyond similar single-center, high-volume bariatric settings remains to be established; multicenter prospective studies with standardized timing, inflammatory profiling, and systematic evaluation of OSA/CPAP adherence are warranted to confirm and generalize these findings.

## Conclusions

In Japanese patients with obesity-related asthma, LSG significantly improved ACT scores, FEV1, and ERV. These results emphasize the importance of recognizing ethnic differences in asthma pathophysiology and provide rare evidence from a Japanese cohort. To the best of our knowledge, this is the first systematic study to demonstrate the beneficial impact of LSG on asthma outcomes in this population. These results, while preliminary, support considering LSG as a therapeutic option for Japanese patients with obesity-related asthma who require substantial weight reduction, pending confirmation in larger multicenter cohorts.

## Figures and Tables

**Figure 3:**
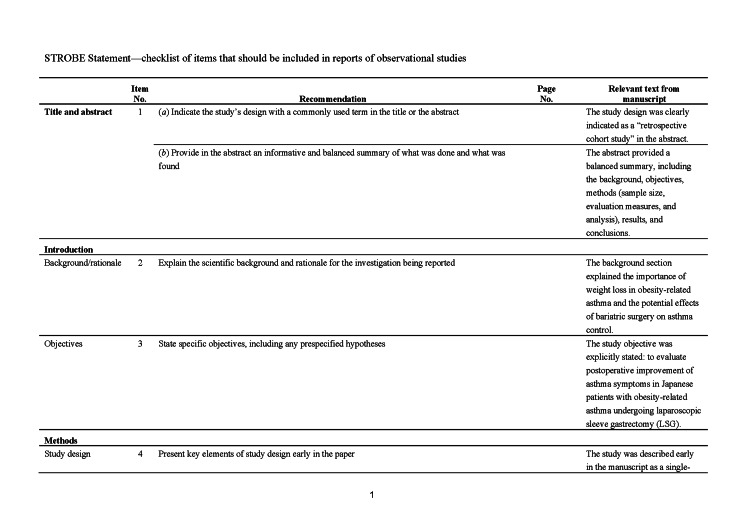
STROBE checklist: part 1 STROBE: STrengthening the Reporting of OBservational studies in Epidemiology

**Figure 4:**
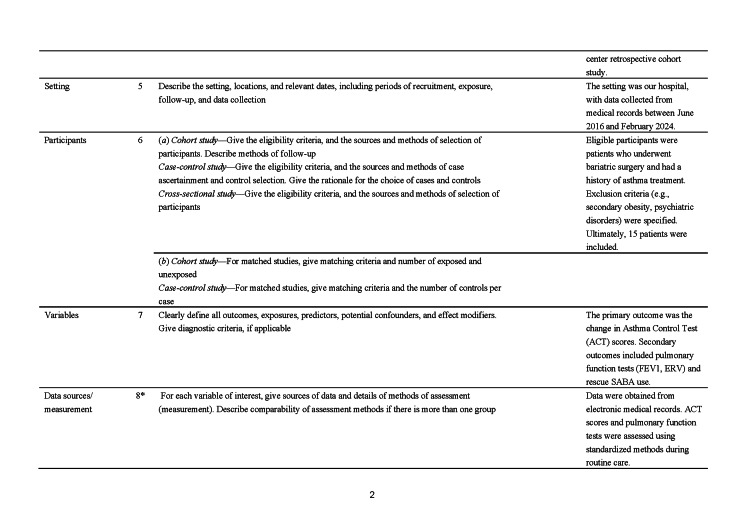
STROBE checklist: part 2 STROBE: STrengthening the Reporting of OBservational studies in Epidemiology

**Figure 5:**
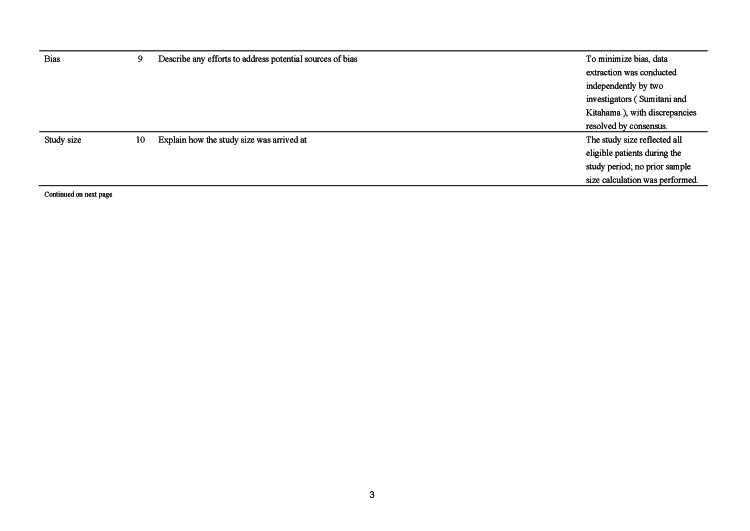
STROBE checklist: part 3 STROBE: STrengthening the Reporting of OBservational studies in Epidemiology

**Figure 6:**
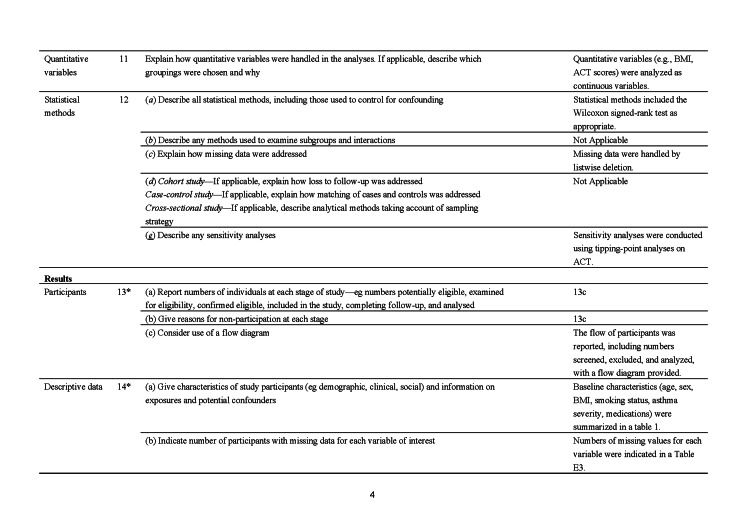
STROBE checklist: part 4 STROBE: STrengthening the Reporting of OBservational studies in Epidemiology

**Figure 7:**
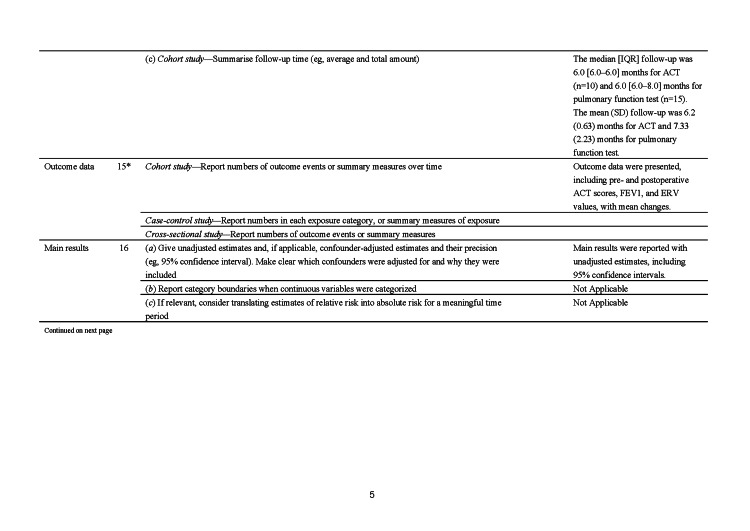
STROBE checklist: part 5 STROBE: STrengthening the Reporting of OBservational studies in Epidemiology

**Figure 8:**
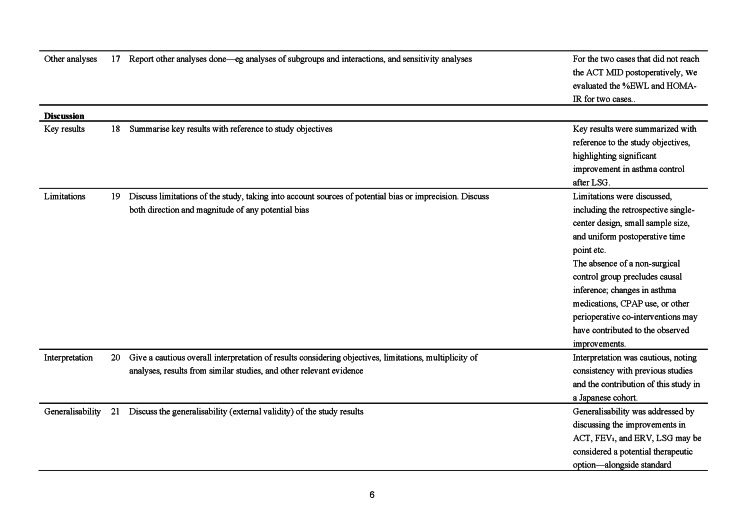
STROBE checklist: part 6

**Figure 9:**
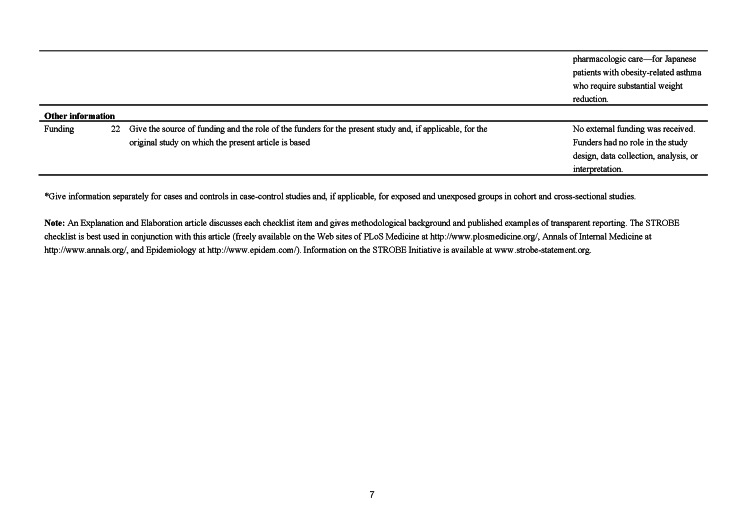
STROBE checklist: part 7

**Figure 10:**
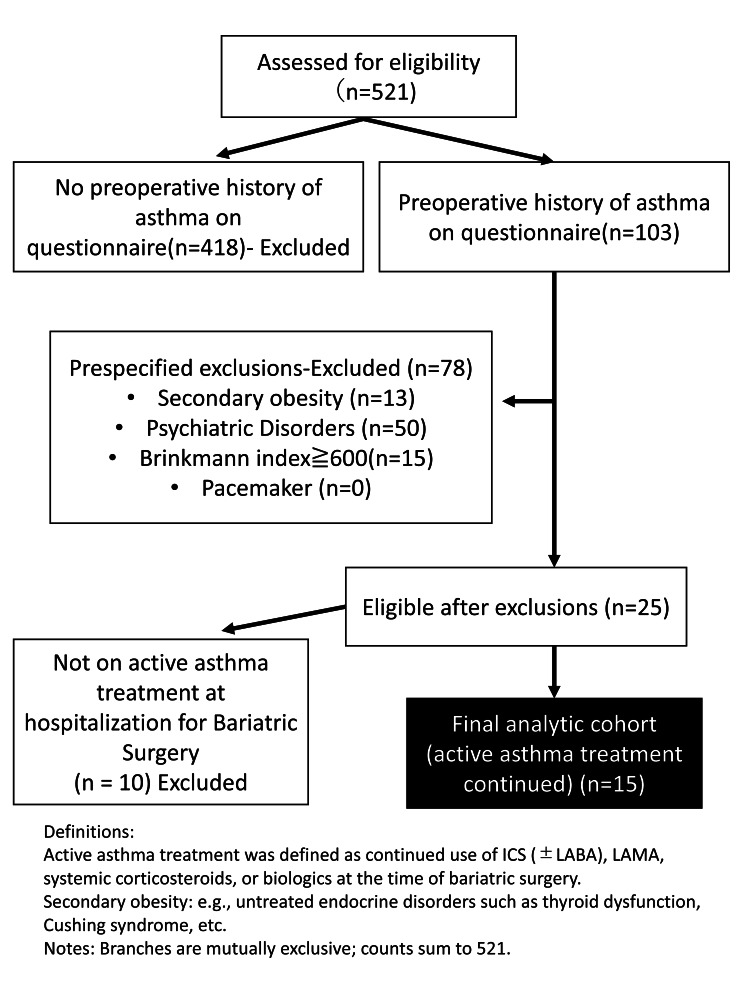
Flow diagram of participants Of the 521 patients who underwent initial bariatric surgery for severe obesity, 103 patients reported a history of bronchial asthma in the preoperative questionnaire, and 15 patients were being treated for bronchial asthma at the time of surgery. ICS: inhaled corticosteroids; LABA: long-acting B2 agonist; LAMA: long-acting muscarinic antagonist

## Appendices

Appendix A 

Appendix B 

Appendix C 

**Table 2:** SABA evaluated based on pre-operative ACT (n=10) To improve readability and clarity, binary indicators were used to represent patient responses. ✓ = selected; ✗ = not selected; ACT: Asthma Control Test; SABA: short-acting B2 agonist

Case	1 or 2 times a day	Several times a week	Less than once a week	No use at all	ACT
					(points)
1	✘	✘	✔	✘	22
2	✘	✘	✔	✘	22
3	✘	✘	✔	✘	22
4	✘	✘	✘	✔	25
5	✘	✘	✔	✘	22
6	✘	✘	✔	✘	23
7	✘	✘	✔	✘	22
8	✘	✔	✘	✘	14
9	✘	✘	✔	✘	22
10	✘	✔	✘	✘	14

Appendix D 

**Table 3:** SABA evaluated based on postoperative ACT (n=10) To improve readability and clarity, binary indicators were used to represent patient responses ✓ = selected; ✗ = not selected; ACT: asthma control test; SABA: short-acting B2 agonist

Case	1 or 2 times a day	Several times a week	Less than once a week	No use at all	ACT
					(points)
1	✘	✘	✘	✔	25
2	✘	✘	✘	✔	25
3	✘	✘	✘	✔	24
4	✘	✘	✘	✔	25
5	✘	✘	✘	✔	25
6	✘	✘	✘	✔	25
7	✘	✘	✘	✔	25
8	✘	✘	✘	✔	25
9	✘	✘	✔	✘	24
10	✘	✘	✔	✘	21

Appendix E 

**Table 4:** Results of various parameters before and after surgery in 15 patients ACT: asthma control test; BMI: body mass index; FEV1: forced expiratory volume in 1 second; ERV: expiratory reserve volume; EWL: excess weight loss; HOMA-IR: homeostasis model assessment-insulin resistance; %EWL: percentage of excess weight loss

CASE	Gender	Age	BMI	Postoperative evaluation period	Pre-operation ACT	Postoperation ACT	Pre-operation eosinophilic count	Post-operation eosinophilic count	Pre-operation IgE	Postoperation IgE	Pre-operation FEV1	Postoperation FEV1	Pre-operation ERV	Postoperation ERV	%EWL at 6months	Pre-operation HOMA-IR
																			Years	kg/m^2^	Months	Points	Points	/μL	/μL	IU/ml	IU/ml	(L)	(L)	(L)	(L)	(%)	
1	Male	53	54.1	6	22	25	269	690	1040	1340	2.9	3.34	0.32	2.38	57.4	6
2	Female	49	55.9	6	22	25	366.3	241.8	100	100	2.56	2.94	0.91	1.41	54.7	5.7
3	Male	40	70.4	6	22	24	331.2	222	2080	3770	2.82	3.6	0.66	1.16	11.7	8.1
4	Female	46	33.2	8	-	-	81.6	141.6	30.9	-	2.4	2.84	0.49	1.47	109.3	5.2
5	Female	41	36.9	6	25	25	69.3	42.7	5	-	2.74	2.8	0.77	1.4	58.5	3.9
6	Female	50	39.4	6	22	25	283.8	299	36.4	-	2.88	2.96	0.89	0.74	63.9	1.6
7	Female	54	36.3	8	23	25	353.8	217.3	725	650	1.96	2.12	0.45	0.81	59.7	1
8	Female	46	46	6	22	25	128.7	151.9	34.2	-	2.14	2.54	0.65	0.94	56.9	4.3
9	Female	57	35.6	6	14	25	288.9	374	296	430	2.3	2.14	0.95	1.23	73	2.9
10	Female	38	61.5	6	22	24	139.5	87.6	35.7	-	2.02	2.22	0.34	0.9	28.5	9.1
11	Female	39	41,02	6	14	21	122.4	97.2	13.8	-	2.58	2.52	0.48	0.51	37.2	3.6
12	Female	37	35	12	-	-	26.1	50.4	-	-	2.9	3.24	1.03	2.21	171.5	2.5
13	Female	55	42.6	10	-	-	110.4	217.6	-	-	3.08	3.4	0.71	1.31	90.2	2.4
14	Female	30	39	12	-	-	376.2	189.6	-	-	2.24	2.58	0.6	1.62	94.2	3.8
15	Female	56	43.6	6	-	-	200.2	138	-	-	1.24	1.76	0.21	0.73	35.9	1.1
